# Effects of interleukin-6 signal inhibition on Treg subpopulations and association of Tregs with clinical outcomes in rheumatoid arthritis

**DOI:** 10.1093/rheumatology/keae196

**Published:** 2024-03-26

**Authors:** Hiroto Yoshida, Mayu Magi, Hiroya Tamai, Jun Kikuchi, Keiko Yoshimoto, Kotaro Otomo, Yoshihiro Matsumoto, Mariko Noguchi-Sasaki, Tsutomu Takeuchi, Yuko Kaneko

**Affiliations:** Product Research Department, Chugai Pharmaceutical Co. Ltd, Kanagawa, Japan; Product Research Department, Chugai Pharmaceutical Co. Ltd, Kanagawa, Japan; Division of Rheumatology, Department of Internal Medicine, Keio University School of Medicine, Tokyo, Japan; Division of Rheumatology, Department of Internal Medicine, Keio University School of Medicine, Tokyo, Japan; Division of Rheumatology, Department of Internal Medicine, Keio University School of Medicine, Tokyo, Japan; Division of Rheumatology, Department of Internal Medicine, Keio University School of Medicine, Tokyo, Japan; Product Research Department, Chugai Pharmaceutical Co. Ltd, Kanagawa, Japan; Product Research Department, Chugai Pharmaceutical Co. Ltd, Kanagawa, Japan; Division of Rheumatology, Department of Internal Medicine, Keio University School of Medicine, Tokyo, Japan; Division of Rheumatology, Department of Internal Medicine, Keio University School of Medicine, Tokyo, Japan

**Keywords:** regulatory T cell, interleukin-6, rheumatoid arthritis, tocilizumab

## Abstract

**Objectives:**

Anti-IL-6 receptor antibodies are clinically efficacious in the management of RA with an associated increase in Tregs; however, the role of functional Treg subsets has yet to be clarified. This study aimed to evaluate how functional Treg subsets are altered by IL-6 receptor blockade and to analyse the relationship between these Treg subsets and the clinical outcome of RA.

**Methods:**

We collected frozen peripheral blood mononuclear cells (PBMCs) from 40 patients with RA who started tocilizumab (TCZ) with or without MTX and 11 healthy controls (HCs). We fractionated Tregs with flow cytometry based on markers of phenotype and function and measured the proportions of detailed Treg subsets sequentially from baseline to week 52.

**Results:**

The proportions of resting Tregs (rTregs) and rTregs+activated Tregs (aTregs) were significantly lower in RA patients at baseline than in HCs. The proportions of all those CD127^low^ Tregs, rTregs, aTregs and rTregs+aTregs were significantly increased with TCZ treatment. In patients treated with TCZ without MTX, rTreg were increased. Patients with an increase in the proportion of rTregs at week 12 had significantly less arthritis flares during the observation period.

**Conclusions:**

Blocking the IL-6 receptor with TCZ increased the proportion of rTregs, a functional Treg subpopulation. Patients with an early increase in rTregs showed a favourable treatment course and this increase in rTregs may reflect molecular remission induced by IL-6 signal inhibition.

## Graphical abstract

**Figure:**
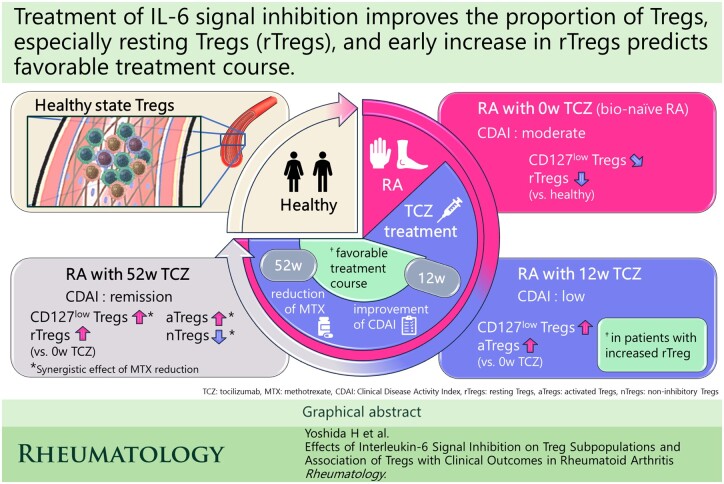

Rheumatology key messages

- IL-6 receptor blockade increased rTregs and aTregs, both of which have the immunosuppressive ability.
- The pure effect of IL-6 receptor blockade was observed as an increase in rTreg.
- Early increase in rTreg cells by IL-6 receptor blockade predicts a favourable treatment course.

## Introduction

RA is a chronic, inflammatory, autoimmune disease that primarily affects the joints [1]. The imbalance between various populations of T cells, including effector T cells (Th1, Th2 and Th17 cells) and Tregs, plays a central role in the initiation and persistence of the chronic inflammation of RA [2, 3].

The role of Tregs in the pathophysiology of RA has been investigated in several studies. In the collagen-induced arthritis (CIA) mouse model, a reduction in the proportion of Tregs among CD4^+^ T cells after treatment with anti-CD25 antibody is associated with arthritis [4], whereas an injection of Tregs improves the symptoms [5]. In humans, genetic polymorphisms of forkhead box P3 (*FOXP3*), a master regulator of Tregs and cytotoxic T-lymphocyte associated protein 4 (*CTLA4*), one of the main co-inhibitory molecules in Tregs, are linked to RA risk [6, 7]. Although there have been many reports investigating the proportion of Tregs in RA patients, the results have been inconsistent and controversial. However, a meta-analysis which defined Treg fraction by a stricter, functionally validated method showed that the proportion of Tregs among CD4^+^ T cells are decreased in RA patients [8]. Therefore, the meta-analysis points to the need for the evaluation of strictly and appropriately defined Tregs.

Human Tregs consist of three subpopulations, each with different phenotypes and functions: CD4^+^CD45RA^+^Foxp3^low^ resting Tregs (rTregs), CD4^+^CD45RA^−^Foxp3^high^ activated Tregs (aTregs) with immunosuppressive activity and CD4^+^CD45RA^−^Foxp3^low^ non-inhibitory Tregs cells (nTregs) without immunosuppressive activity [9]. This definition of Treg subpopulations provides us with an accurate and functional classification of Tregs, which may allow a more accurate assessment of the proportions of Tregs in RA.

IL-6 is a pro-inflammatory cytokine that plays an important role in the pathophysiology of RA, including inflammatory cell migration, angiogenesis and joint destruction [10]. In addition, IL-6 also contributes to the induction and maintenance of the autoimmune process in RA through its action on T cell development and B cell maturation. In particular, IL-6 affects the differentiation of T cells into effector T cells and Tregs [11]. Specifically, IL-6 was also shown to decrease Foxp3 expression and increase RORγt expression [12]. These effects of IL-6 on T cells may adversely affect the Th17/Treg balance in RA patients [13, 14].

The anti-IL-6 receptor antibodies, tocilizumab (TCZ) and sarilumab, are shown to be clinically effective against RA [15, 16]. IL-6 receptor blockade has been shown to improve many aspects of RA symptoms and to alter the proportions of T cell subsets. In non-clinical studies, it has been reported that IL-6 receptor antibodies suppressed differentiation into Th17 cells [17–19] and induced differentiation toward the Treg subpopulation [20]. In addition, there are several clinical reports that blocking the IL-6 receptor decreases Th17, and increases Tregs in peripheral blood in correlation with a decrease in disease activity, suggesting the mechanism of the effectiveness of IL-6 signal inhibition in the disease [13, 21, 22]. Although Treg should be classified strictly and appropriately, there has been no detailed analysis from the perspective of Treg subpopulations in RA, and it is not clear how Treg subpopulations are affected by IL-6 receptor blockade or how changes in Treg subpopulations affect the clinical outcome of RA.

In this study, we aimed to define in detail the impact of IL-6 on Treg subpopulations by evaluating how functionally distinct Treg subpopulations are altered by IL-6 receptor blocking with TCZ in bio-naïve RA patients. We also aimed to clarify the significance of changes in the proportion of Treg subpopulations in RA by analysing the relationship between the alteration in subpopulations of Treg with TCZ and clinical outcomes in patients with RA.

## Methods

### Patients

We collected peripheral blood from 40 bio-naïve RA patients who started TCZ as their first biologic DMARD between October 2011 and December 2014 and from 11 healthy controls (HCs). All subjects provided their written informed consent. All patients were diagnosed with RA based on the 1987 revised criteria of the ACR for the classification of RA or the 2010 ACR/ EULAR classification criteria [23, 24]. TCZ were administered either with or without the conventional synthetic DMARD (csDMARD), including MTX, according to the attending physicians’ decision. We confirmed that the HCs did not have any autoimmune disease, severe allergic disorder, malignancy or infection.

The study protocol was approved by the ethics committees of Keio University School of Medicine (#20140479) and the Ethical Review Committee of Chugai Pharmaceutical Co. Ltd (Approval No.: C102). It was carried out in accordance with the principles of the Declaration of Helsinki. All participants provided written informed consent prior to enrolment in the study.

### Clinical assessments and evaluation of efficacy

Demographic and clinical characteristics were obtained from the patients’ medical records; age, sex, disease duration, use of MTX, dose of MTX, use of glucocorticoids (GCs), dose of GCs, the levels of CRP, MMP-3, RF and anticyclic citrullinated peptide antibody (ACPA), seropositive, ESR, value; 28-joint disease activity score calculated with CRP (DAS28-CRP), 28-joint disease activity score calculated with ESR (DAS28-ESR), Simplified Disease Activity Index (SDAI), Clinical Disease Activity Index (CDAI) and HAQ Disability Index (HAQ-DI) score. RF-positive and ACPA-positive were defined as the elevation of RF (>15 IU/ml) and ACPA (≥4.5 U/ml). Seropositive was defined as RF-positive and/or ACPA-positive. The cut-off values for remission, low disease activity (LDA), moderate disease activity (MDA) and high disease activity (HDA) were classified based on CDAI as follows: remission, CDAI ≤ 2.8; LDA, 2.8 < CDAI ≤ 10; MDA, 10 < CDAI ≤ 22; and HDA, CDAI > 22 [25]. Disease flare was defined as an increase in the categories.

### Flow cytometry staining and data acquisition

We collected peripheral blood at baseline, weeks 12, 24 and 52 after TCZ initiation from patients and once from HCs. Peripheral blood mononuclear cells (PBMCs) were separated by density gradient with Ficoll-Paque Plus (GE Healthcare, Uppsala, Sweden) and cryopreserved at -80°C in CELLBANKER 1 (Nippon Zenyaku Kogyo, Fukushima, Japan) until use. Thawed cells were treated with FcR Blocking Reagent (Miltenyi Biotec, Bergisch Gladbach, Germany) and stained for 30 min at 4°C under darkened conditions with the following fluorophore-labelled mAbs: anti-CD4-APC/Cyanine7 (RPA-T4), and anti-CD25-Brilliant Violet 421 (BC96), anti-CD45RA-Alexa Fluor 488 (HI100), anti-CD127(IL-7Rα)-PerCP/Cyanine5.5 (A019D5) from BioLegend (San Diego, CA, USA); and LIVE/DEAD Fixable Aqua Stain from Thermo Fisher Scientific Inc. (Waltham, MA, USA). Cells were incubated for 1 h at 4°C with eBioscience Transcription Factor Fix/Perm Buffer (Thermo Fisher Scientific Inc.) and stained for 30 min at 4°C with intracellular antibody anti-FOXP3-PE (259D, BioLegend) under darkened conditions. Data from stained cells were acquired on a MACSQuant analyser (Miltenyi Biotec).

### Flow cytometry data analysis and treg subsets

Flow cytometry data were analysed using FlowJo 10.5.3, and the following subsets of Tregs were analysed: CD127^low^ Tregs (CD4^+^CD25^+^CD127^low^, the population of Tregs as a whole), resting Tregs (rTregs; CD4^+^CD45RA^+^Foxp3^low^, naïve type highly proliferative Tregs with immunosuppressive ability, activated Tregs (aTregs; CD4^+^CD45RA^−^Foxp3^high^, memory type long-living Tregs with immunosuppressive ability), non-inhibitory Tregs (nTregs; CD4^+^CD45RA^−^Foxp3^low^, cytokine-secreting non-suppressive Tregs). The gating strategy for these Treg subsets is shown in Supplementary Fig. S1, available at *Rheumatology* online.

### Statistical analysis

Data are presented as median and interquartile range (IQR). Continuous variables and categorical data were compared with the Wilcoxon signed-rank test and Fisher’s exact test, respectively. The correlation was analysed using the Spearman rank correlation coefficient. *P* values of <0.05 were considered statistically significant. All statistical analyses were performed with JMP 15.0.0 (SAS Institute, Cary, NC, USA).

## Results

### Baseline characteristics of patients

Table 1 shows the baseline demographics and clinical characteristics of the enrolled RA patients (*n *=* *40) and HCs (*n *=* *11). RA patients were older and female-dominant compared with HCs.

**Table 1.** Clinical characteristics of RA patients and HCs at baseline

Clinical characteristics	RA	HCs
	(*n *=* *40)	(*n *=* *11)
Age, years	60.0 (50.3–64.8)	45.0 (35.0–56.0)
Female	35 (87.5)	7 (63.6)
Disease duration, years	1.5 (0.5–6.3)	
Usage of MTX	28 (70.0)	
Dose of MTX in MTX-use patients, mg/week	10.0 (8.0–12.0)	
Usage of GCs	9 (22.5)	
Dose of GCs in GCs-use patients, mg/day	5.0 (3.0–8.0)	
DAS28-CRP	3.7 (3.2–4.6)	
DAS28-ESR	4.7 (3.8–5.2)	
SDAI	16.8 (12.6–24.7)	
CDAI	16.3 (12.2–24.5)	
HAQ-DI	0.8 (0.3–1.1)	
CRP, mg/dL	0.5 (0.1–1.2)	
ESR, mm/h	35.5 (16.3–53.0)	
MMP-3, ng/mL	81.1 (45.7–130.0)	
RF-positive	31 (77.5)	
ACPA-positive	31 (77.5)	
Seropositive	34 (85.0)	

Continuous data are expressed as a median (interquartile range) and categorical data as numbers (percentages).

ACPA: anticyclic citrullinated peptide antibody; CDAI: Clinical Disease Activity Index; DAS28: 28-joint disease activity score; GCs: glucocorticoids; HAQ-DI: Health Assessment Questionnaire Disability Index; IQR: interquartile range; SDAI: Simplified Disease Activity Index.

### Proportions of Treg subpopulations in bio-naïve RA patients and HCs

In bio-naïve RA patients, the proportion of rTregs among CD4^+^ T cells was negatively correlated with DAS28-ESR and RF, and the proportion of aTregs was positively correlated with MMP-3. The proportion of rTregs+aTregs, Treg subsets having immunosuppressive ability, showed a significantly positive correlation with MMP-3. No other baseline Treg subsets significantly correlated with disease activity or serum markers (Supplementary Table S1, available at *Rheumatology* online).

**Figure 1.**
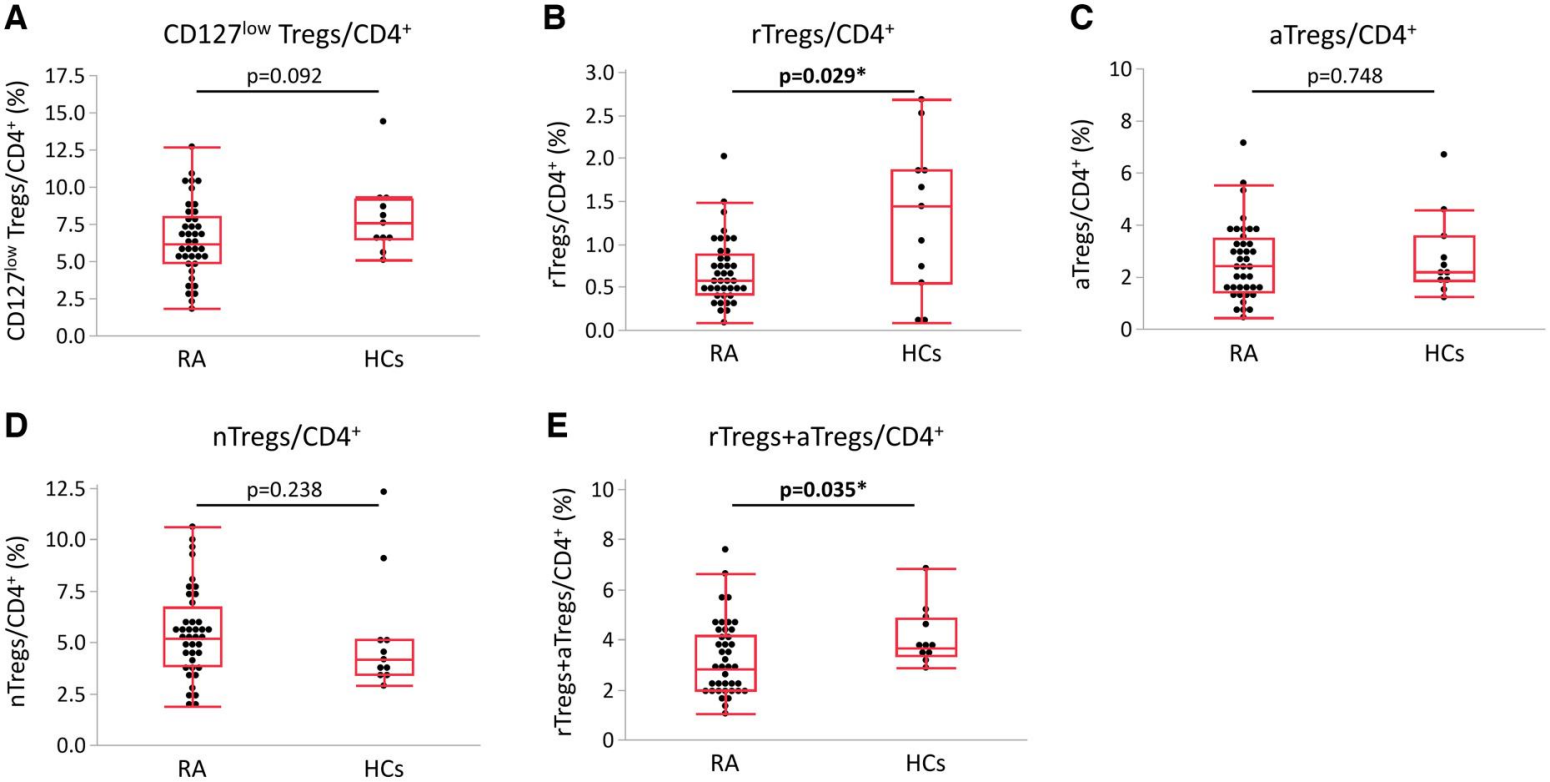
Comparison between proportions of Treg subpopulations in RA patients and HCs. Percentages of (A) CD127^low^ Tregs, (B) rTregs, (C) aTregs, (D) nTregs and (E) rTregs+aTregs among CD4^+^ cells at week 0 were compared between RA patients (RA) and healthy controls (HCs). The bottom, middle and upper lines of the box plots correspond to the 25th, 50th and 75th percentiles, respectively. Data were analysed by the Wilcoxon signed-rank test. ^*^Statistically significant difference (*P* < 0.05)

Comparison of CD127^low^ Tregs representing the whole population and each Treg subset (rTregs, aTregs and nTregs) between RA patients and HCs showed that the proportion of CD127^low^ Treg at baseline tended to be lower in RA patients than in HCs, but not significant (Fig. 1A), whereas the proportions of the rTreg subpopulation were significantly lower in RA patients (Fig. 1B–D). The proportion of rTregs+aTregs was also significantly lower in RA patients than in HCs (Fig. 1E).

### Change in disease activity score during IL-6 receptor blockade

During the treatment of IL-6 receptor blockade with TCZ, disease activity was significantly improved. The median CDAI was 16.3 before treatment (baseline) and decreased to 7.6, 3.3, 1.5 and 1.8 at weeks 4, 12, 24 and 52, respectively (Supplementary Fig. S2, available at *Rheumatology* online).

### Changes in proportions of treg subsets during IL-6 receptor blockade

We next looked at how the proportions of the Treg subsets changed over time with TCZ.

**Figure 2.**
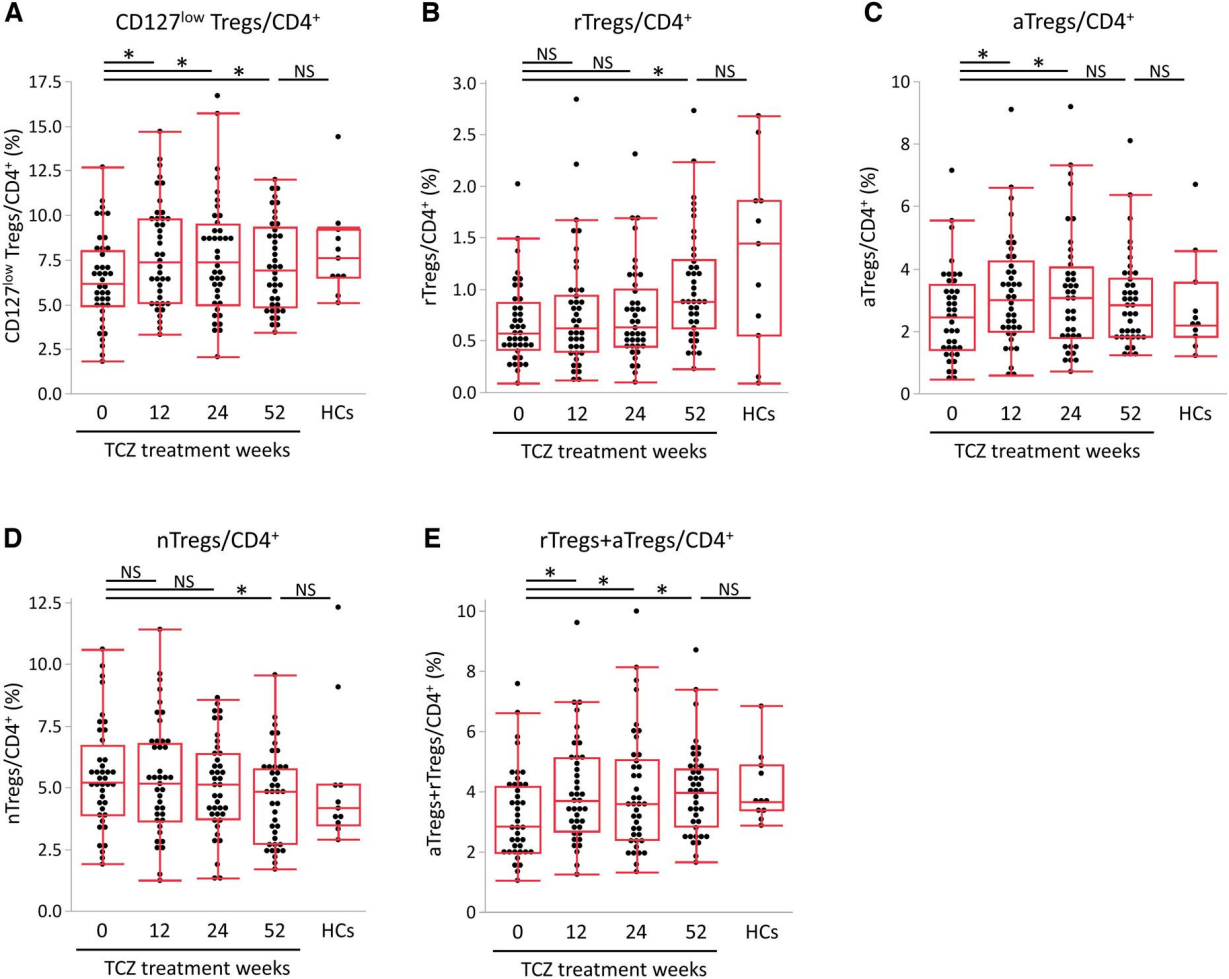
Chronological changes in proportions of Treg subsets during TCZ treatment. Chronological changes in the percentages of (A) CD127^low^ Tregs, (B) rTregs, (C) aTregs, (D) nTregs and (E) rTregs+aTregs relative to CD4^+^ cells from baseline (week 0) to week 52 of TCZ treatment are shown. The bottom, middle and upper lines of the box plots correspond to the 25th, 50th and 75th percentiles, respectively. Changes in proportions of Treg subsets during TCZ treatment were analysed by Wilcoxon matched-pairs signed-rank test (*vs* week 0). Comparisons between RA patients at week 52 of TCZ treatment and HCs were analysed by the Wilcoxon signed-rank test. ^*^Statistically significant difference (*P* < 0.05)

The CD127^low^ Tregs was significantly increased at week 12 and remained increased until week 52 (Fig. 2A) compared with baseline. Regarding each subset, the subsets of rTregs and aTregs were significantly increased at week 52 and weeks 12 and 24, respectively (Fig. 2B and C), whereas the subset of nTregs was significantly decreased at week 52 (Fig. 2D). The subset of rTregs+aTregs remained increased from week 12 to week 52 (Fig. 2E). The proportions of any Treg subsets at week 52 became similar to that of HCs (Fig. 2).

### Effect of concomitant drugs on Treg subsets

**Figure 3.**
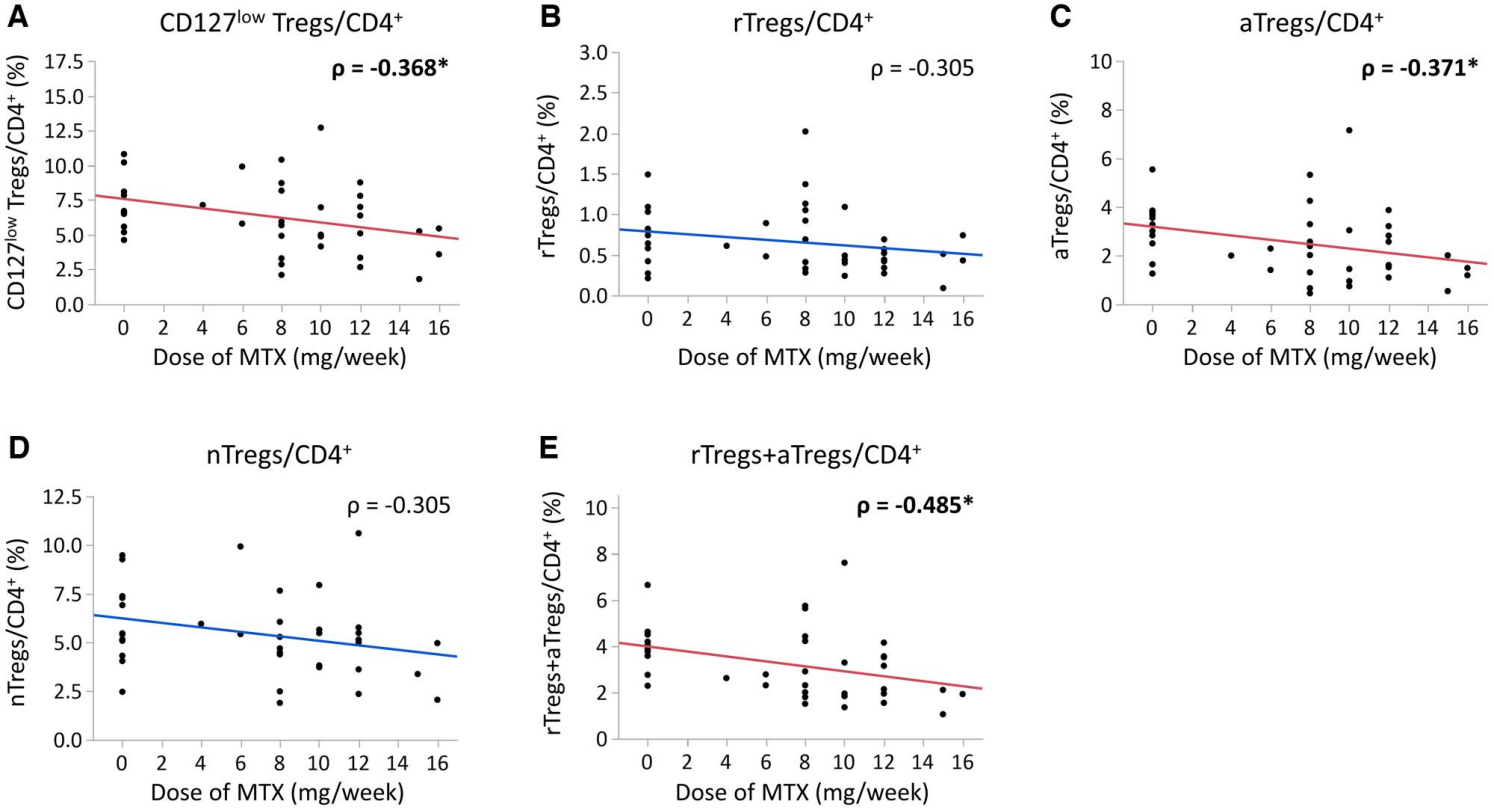
Correlations at baseline between the proportions of Treg subsets and the dose of MTX. The relationships at week 0 between MTX dose and the proportions of (A) CD127^low^ Tregs, (B) rTregs, (C) aTregs, (D) nTregs and (E) rTregs+aTregs relative to CD4^+^ cells are shown. Data were analysed by the Spearman rank correlation coefficient, and Spearman’s ρ values are provided. *Statistically significant correlations (*P* < 0.05)

To determine the effect of concomitant drugs, MTX and prednisolone (PSL), on Treg subsets, the relationship between the dose of MTX or PSL and the proportion of each of the Treg subsets at baseline was examined. The proportions of aTregs and rTregs+aTregs were significantly lower in patients treated with MTX than those in patients without MTX (Supplementary Fig. S3, available at *Rheumatology* online), and the dose of MTX was negatively correlated with the proportions of CD127^low^ Tregs, aTregs and rTregs+aTregs significantly (Fig. 3). On the other hand, the dose of PSL did not correlate with any Treg subsets (Supplementary Fig. S4, available at *Rheumatology* online).

### Changes in proportions of Treg subsets during IL-6 receptor blockade with or without MTX

Since changes in the proportions of Treg subsets (particularly CD127^low^ Tregs, aTregs and rTregs+aTregs) may be affected by MTX dose, we investigated the pure effect of TCZ treatment on Treg subsets by stratifying patients according to their use of MTX; MTX used group (*n *=* *28) and MTX non-used group (*n *=* *12) (Supplementary Table S2, available at *Rheumatology* online).

**Figure 4.**
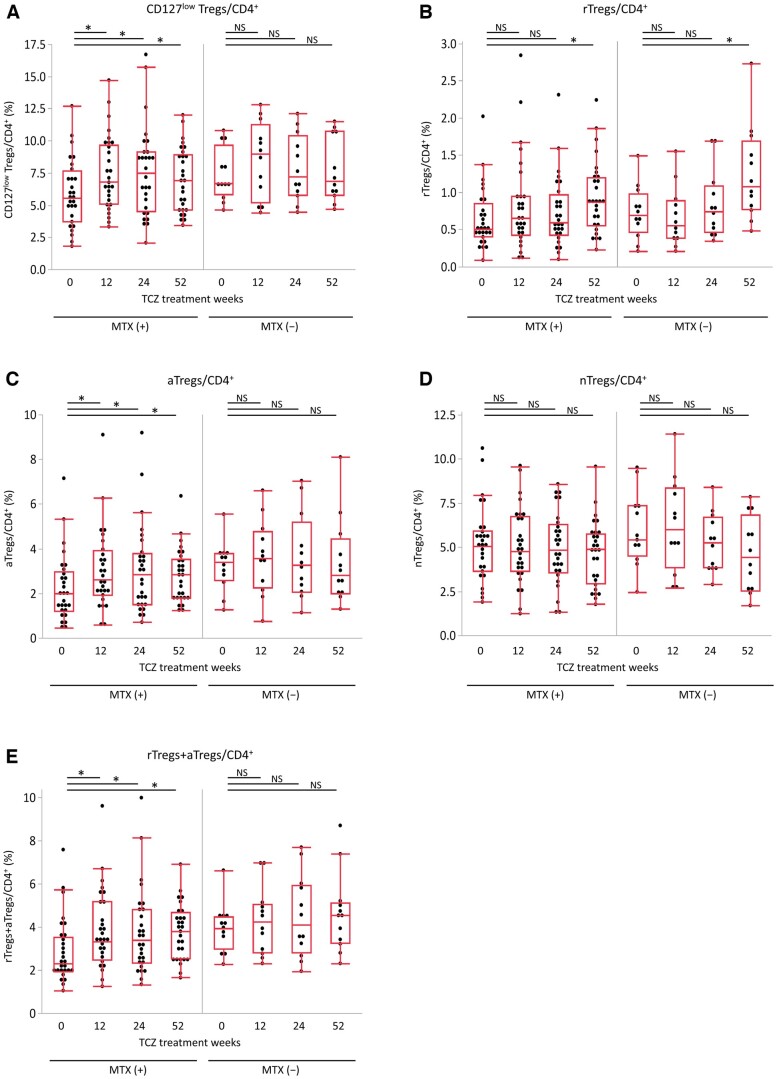
Changes in proportions of Treg subsets during TCZ treatment with or without MTX. Chronological changes in the proportions of (A) CD127^low^ Tregs, (B) rTregs, (C) aTregs, (D) nTregs and (E) rTregs+aTregs relative to CD4^+^ cells from baseline (week 0) to week 52 of TCZ treatment are presented separately for RA patients using MTX and those not using MTX. The bottom, middle and upper lines of the box plots correspond to the 25th, 50th and 75th percentiles, respectively. Data were analysed by Wilcoxon matched-pairs signed rank test (*vs* week 0). ^*^Statistically significant difference (*P* < 0.05)

In the MTX used group, TCZ significantly increased the proportions of CD127^low^ Tregs, rTregs, aTregs and rTregs+aTregs compared with those at baseline (Fig. 4). Of note, during this observational period, MTX dose was decreased from a median of 10.0 mg/week (IQR: 8.0–12.0 mg/week) at baseline to a median of 4.0 mg/week (IQR: 0.0–6.0 mg/week) at week 52. On the other hand, in the MTX non-used group, which represented the pure effect of TCZ treatment, the population of rTregs at week 52 was increased (Fig. 4), suggesting the proportion of rTregs was increased with TCZ itself.

### Relationship between changes in rTregs and disease activity

**Figure 5.**
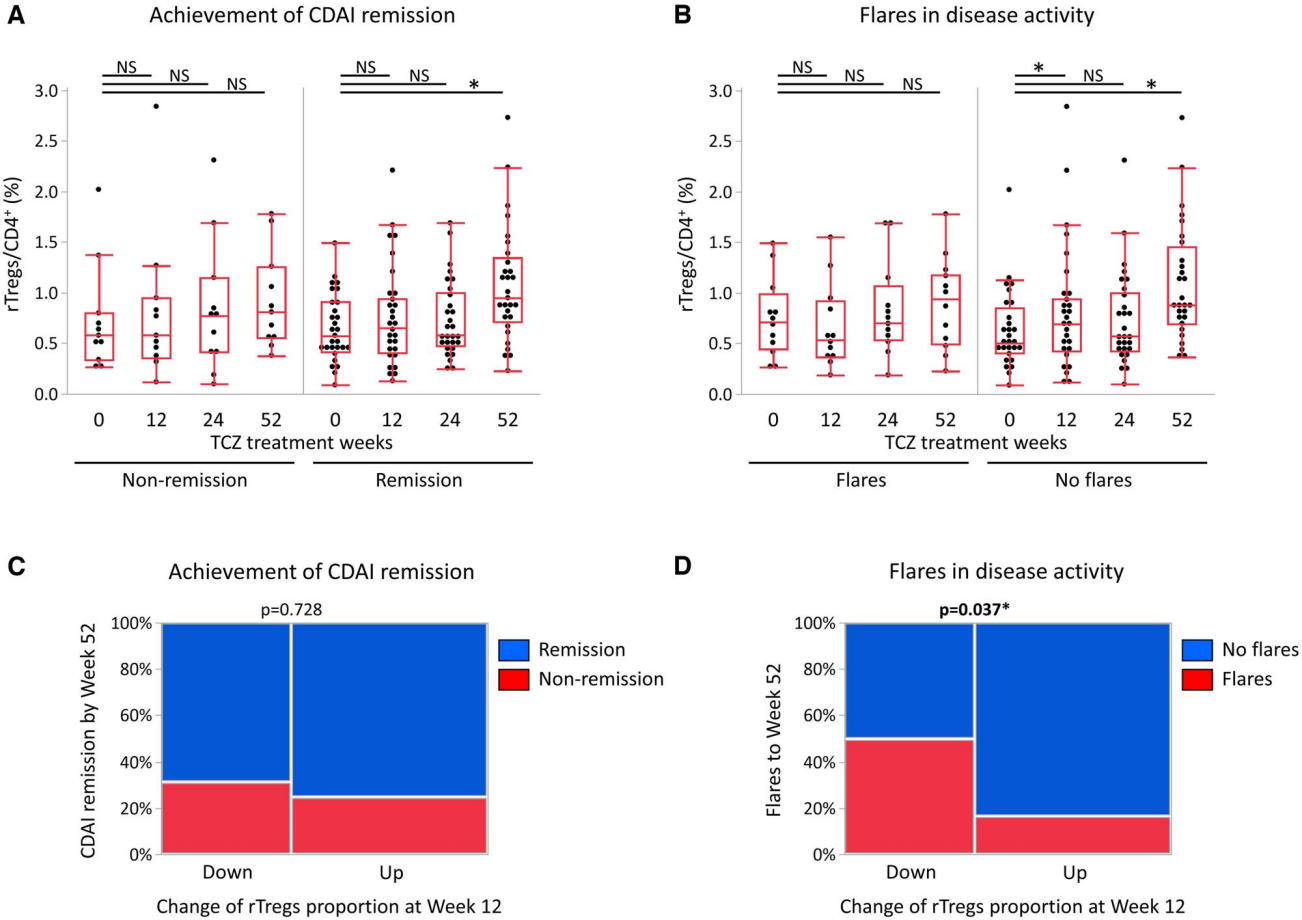
Relationship between change in the proportion of rTregs and clinical response with TCZ treatment. Chronological change in the proportion of rTregs is presented separately for RA patients with and without (A) achieved remission, (B) flare of disease activity. Changes in the proportion of rTregs at week 12 were used to predict, (C) achievement of CDAI remission and (D) the presence or absence of flares. The bottom, middle and upper lines of the box plots correspond to the 25th, 50th and 75th percentiles, respectively. Data were analysed by Wilcoxon matched-pairs signed rank test (*vs* week 0) (A, B) or Fisher’s exact test (C, D). ^*^Statistically significant difference (*P* < 0.05)

To investigate the role of rTregs in the clinical outcome of RA, we evaluated changes in rTregs with respect to CDAI remission achievement and disease flare. When we divided patients into two groups according to the CDAI remission achievement at least once through 52 weeks, the proportion of rTregs was increased in patients who achieved remission (*n *=* *29) but not increased in those who did not achieve remission (*n *=* *11) (Fig. 5A).

When we divided patients according to the presence (*n *=* *12) or absence (*n *=* *28) of disease flare during the observation period, the proportion of rTregs was significantly increased in the group without flares (Fig. 5B).

We further examined whether early changes in the proportion of rTregs could predict the future course of RA. The increase or decrease in the proportion of rTregs at week 12 relative to the proportion at week 0 did not associate with CDAI remission achievement by week 52 (Fig. 5C). On the other hand, patients with an increase in the proportion of rTregs at week 12 had significantly lower rates of flares during the 52 weeks observation period than those with a decrease in the proportion of rTregs (Fig. 5D).

## Discussion

This study demonstrated that rTregs were increased by blocking the IL-6 receptor, and all other Treg subsets except nTregs were increased with dose reduction of concomitant MTX. An increase in rTregs along with TCZ was associated with remission achievement and the absence of disease flare, and an early increase in rTregs predicted a favourable clinical course with TCZ.

Miyara *et al.* reported that within the whole population of Tregs, there are several Treg subpopulations whose functions differ [9]. In particular, among these Treg subpopulations, nTregs are reported not only to lack immunosuppressive ability but also to produce pro-inflammatory cytokines such as IL-17 and IFN-γ. In our study, the subpopulations of rTregs and aTregs, both of which are considered to have the immunosuppressive ability, were increased along with TCZ treatment, and conversely, nTregs were decreased, demonstrating the increase in Tregs with IL-6 receptor blockade was ascribed to a proportional increase in the subsets of immunosuppressive Tregs and this is one of the mechanisms of the effectiveness of IL-6 receptor blockade. Although the effect of MTX on the increase in immunosuppressive Tregs cannot be ruled out in our study, a proportional increase in aTregs has also been described in patients with giant cell arteritis (GCA) treated with TCZ [26], indicating that increases in immunosuppressive Tregs are a universal effect of IL-6 receptor blockade in various diseases beyond RA.

In this study, TCZ treatment changed the proportion of all Treg subsets to HCs levels, but RA patients were significantly older and tended to be more dominant with women than HCs. Since it has been reported that there is no significant change in Treg proportion in people aged 20 years and older but Treg proportion is lower in women than men [27–30], our study suggested that, TCZ treatment has the potential to improve Treg proportion in patients with RA to HCs levels.

The analysis focusing on patients treated with TCZ without MTX in this study showed that the increase was only observed in rTregs, suggesting the increase in rTregs is absolutely the pure effect of IL-6 receptor blockade. And the increase in rTregs became significant at week 52 for the first time in our study. Therefore, if we had observed longer than 52 weeks, an increase in aTregs might have also been captured because rTregs have the properties of naïve CD4^+^ T cells and become aTregs as differentiation proceeds [9]. The findings that the Treg subpopulations elevated by IL-6 receptor blockade were subpopulations with immunosuppressive ability was a key finding of this study, also suggesting that IL-6 is involved in a reduction in the proportions of immunosuppressive Tregs.

In this study, the proportion of some Treg subsets was inversely correlated with the dose of MTX, and the proportion of CD127^low^ Tregs were increased synergistically by TCZ induction and the decrease in MTX dose. Clinical efficacy of TCZ is higher with concomitant MTX than that of TCZ monotherapy in the early phase of TCZ use, however, it becomes comparable between TCZ monotherapy and TCZ with concomitant MTX after 1 year of TCZ therapy [31]. Therefore, TCZ is recommended to be started with MTX but can be altered as monotherapy with MTX tapering in clinical practice. Our study shows that such a treatment strategy is reasonable from the viewpoint of raising CD127^low^ Tregs.

Our study also showed that rTregs were elevated in TCZ-treated patients who achieved clinical remission or who had a stable treatment course without flares. We also found that flares were less likely to occur in TCZ-treated patients who had an early elevation of rTregs. On the other hand, it should be noted that changes in rTregs at week 12 had no impact on the remission rates by week 52, indicating that even patients without early rTreg elevation are not less likely to respond to TCZ. In other words, patients with decreased rTregs at week 12 may have had some flares, but they eventually achieved remission. These results suggest that early changes in rTregs by IL-6 receptor antibody may predict the course of treatment but not the outcome. Even patients with an early decrease in rTregs may be able to achieve a favourable treatment outcome without changing IL-6 receptor antibody to other medications.

We focused on Tregs in this study, but it was reported that IL-6 receptor antibody not only increases Treg proportion but also changes the proportion of other T cell subsets [13, 32]. Furthermore, considering reports that IL-6 receptor antibody brings not only T cells but also other subsets closer to HCs levels overall [33–35], the relationship between increased rTregs and a favourable treatment course may provoke the idea that this is due to TCZ leading to normalization of molecular expression (molecular remission). In particular, rTregs were significantly less prevalent in bio-untreated and active patients than in HCs. Thus, an increase in this Treg subpopulation to a level similar to that in HCs may be evidence of approaching molecular remission. In addition, an increase in this Treg subpopulation with the characteristics of naïve CD4^+^ T cells may represent a change from a pathological immune state towards a normal immune state, suggesting that patients with elevated rTregs can be considered to be close to molecular remission, and a result, a favourable treatment course could be maintained. Therefore, these findings suggest that Treg elevation by IL-6 receptor antibody is an element of molecular remission. In contrast, there was no significant change in the proportion of rTregs with TCZ treatment, although patients who did not achieve CDAI remission or experienced flare may not have achieved sufficient statistical power. These results may be reasonable given our discussion that an increase in rTregs is one indicator of molecular remission.

It has been suggested that RA patients, especially those with active RA, have a reduced Treg suppressive function compared with HCs [36]. One limitation of this study was that we only observed the changes in the proportions of Treg subsets, and we did not examine the molecular mechanisms of IL-6 receptor antibody: the effects of IL-6 receptor blockade on the differentiation and proliferation of each Treg subsets or on the ability of Tregs to suppress the immune response. Although it has been reported that IL-6 inhibits Treg differentiation, it remains unclear which Treg subpopulations are inhibited. It has been reported that IL-6 converted Tregs, especially thymus-derived natural Tregs to Th17 cells [37, 38]. In addition, natural Tregs with a naïve phenotype has been shown to be more responsive to IL-6 due to higher expression of IL-6 receptors than inducible Tregs with a memory phenotype, which may explain the mechanism by which IL-6 signal inhibition increased the number of rTregs. Regarding the effect of IL-6 on Treg function, some reports suggested that Treg-mediated suppression of T cell proliferation was reduced in the presence of IL-6, which is the result of IL-6 affecting effector T cells but not Tregs [39, 40]. In addition, one reported mechanism by which Tregs lose their suppressive function is through the expression of a variant of Foxp3 lacking exon 2 (Foxp3Δ2) [41]. It has been reported that the increased proportion of Foxp3Δ2 cells in active GCA patients decreases with TCZ treatment [26, 42]. In contrast, Treg suppressive activity as assessed by immunosuppression assay, which is reduced in active GCA compared with HCs, was not restored by TCZ treatment [26]. Therefore, regarding the mechanism by which IL-6 affects Tregs, IL-6 is likely to change the proportion of Tregs by affecting the differentiation and conversion of Tregs, but the effect of IL-6 on Treg function remains an open question.

In this study, we evaluated only IL-6 receptor antibody, but current RA therapies also use agents that target molecules such as tumour necrosis factor (TNF)-α and Janus kinase (JAK). Some clinical reports show that TNF-α inhibitors increased Treg proportion and JAK inhibitors did not change or decrease Treg proportion [43–45]. On the other hand, *in vitro* studies have shown that TNF-α induced Treg differentiation and JAK inhibitors suppressed Treg differentiation [44, 46, 47]. From these molecular mechanisms, the increase in Tregs in clinical practice with TNF-α inhibitors may be the result of indirect effects, such as a decrease in IL-6 production. These results suggest that although various drugs target different molecules in RA, Treg-increasing effects may be a feature of IL-6 receptor antibody. In summary, we demonstrated that IL-6 receptor blockade with TCZ increased the proportion of rTregs in patients with RA. rTregs were significantly increased in patients who achieved clinical remission without flares during the observation period and patients whose rTregs were increased early after IL-6 receptor blockade initiation had a favourable treatment course. These findings may reflect the molecular remission induced by IL-6 receptor blockade.

## Supplementary Material

keae196_Supplementary_Data

## Data Availability

The data underlying this article will be shared on reasonable request to the corresponding author.
