# Supplementary material for: Effects of interleukin-6 signal inhibition on Treg subpopulations and association of Tregs with clinical outcomes in rheumatoid arthritis
Source: Rheumatology (Oxford). 2024 Mar 26;63(9):2515–24. doi: 10.1093/rheumatology/keae196 (PMC11371379; doi:10.1093/rheumatology/keae196)
Supplement: keae196_Supplementary_Data [file keae196_supplementary_data.docx]

SUPPLEMENTARY MATERIAL

**Supplementary Table S1. Correlations between proportions of Treg subsets and composite measures of disease activity, Health Assessment Questionnaire-Disability Index score, and serum markers at baseline**

|  | DAS28-CRP | DAS28-ESR | SDAI | CDAI | HAQ-DI | CRP | ESR | MMP-3 | RF | ACPA |
| --- | --- | --- | --- | --- | --- | --- | --- | --- | --- | --- |
| CD127^low^ Tregs / CD4^+^ | 0.086 | 0.051 | 0.118 | 0.121 | 0.092 | 0.050 | 0.051 | 0.215 | −0.247 | −0.011 |
| rTregs / CD4^+^ | −0.231 | **−0.329*** | −0.275 | −0.258 | −0.093 | −0.006 | −0.272 | 0.153 | **−0.424*** | −0.223 |
| aTregs / CD4^+^ | 0.149 | 0.035 | 0.238 | 0.235 | 0.046 | 0.137 | 0.011 | **0.333*** | −0.152 | 0.067 |
| nTregs / CD4^+^ | 0.103 | 0.161 | 0.071 | 0.051 | −0.004 | 0.236 | 0.219 | 0.171 | 0.017 | 0.082 |
| rTregs+aTregs / CD4^+^ | 0.095 | −0.048 | 0.176 | 0.179 | 0.036 | 0.102 | −0.092 | **0.370*** | −0.264 | 0.011 |

Correlations between the proportions of Treg subsets and the clinical characteristics at Week 0 are shown. Data were analyzed by Spearman rank correlation coefficient, and Spearman’s ρ values are provided. *, Statistically significant correlation (*P* value < 0.05)

**Supplementary Table S2. Clinical characteristics of RA patients using/not using MTX at baseline**

| **Clinical Characteristics** | **Using MTX**  **(*n*=28)** | **Not using MTX**  **(*n*=12)** | ***P* value** |
| --- | --- | --- | --- |
| Age, years | 60.0 (49.5 to 64.0) | 60.5 (51.5 to 65.0) | 0.745 |
| Female | 26 (92.9) | 9 (75.0) | 0.149 |
| Disease duration, years | 2.0 (0.5 to 10.5) | 1.0 (0.2 to 5.3) | 0.140 |
| Usage of MTX | 28 (100.0) | 0 (0.0) | **<0.0001*** |
| Dose of MTX, mg/week | 10.0 (8.0 to 12.0) | 0.0 (0.0 to 0.0) | **<0.0001*** |
| Usage of GCs | 4 (14.3) | 5 (41.7) | 0.097 |
| Dose of GCs in GCs-use patients, mg/day | 3.0 (2.3 to 4.5) | 6.0 (5.5 to 10.0) | **0.025*** |
| DAS28-CRP | 3.6 (3.1 to 4.3) | 3.8 (3.5 to 5.3) | 0.309 |
| DAS28-ESR | 4.6 (3.7 to 5.1) | 4.8 (3.8 to 6.3) | 0.575 |
| SDAI | 15.5 (11.5 to 21.5) | 20.4 (14.0 to 33.3) | 0.210 |
| CDAI | 15.3 (11.1 to 20.1) | 19.0 (12.8 to 31.2) | 0.275 |
| HAQ-DI | 0.8 (0.3 to 1.1) | 0.7 (0.0 to 1.6) | 0.965 |
| CRP, mg/dl | 0.2 (0.1 to 0.8) | 0.7 (0.4 to 3.4) | 0.069 |
| ESR, mm/h | 28.5 (15.3 to 46.0) | 54.0 (20.0 to 80.5) | 0.114 |
| MMP-3, ng/mL | 49.9 (42.1 to 99.4) | 129.4 (95.7 to 196.3) | **0.001*** |
| RF-positive | 24 (85.7) | 7 (58.3) | 0.097 |
| ACPA-positive | 26 (92.9) | 5 (41.7) | **0.001*** |
| Seropositive | 26 (92.9) | 8 (66.7) | 0.055 |

Continuous data are expressed as a median (interquartile range) and categorical data as numbers (percentages). Data were analyzed by Wilcoxon signed rank test (continuous data) or Fisher's exact test (categorical data). *, Statistically significant correlation (*P* value < 0.05)

ACPA, anticyclic citrullinated peptide antibody; CDAI, Clinical Disease Activity Index; CRP, C-reactive protein; DAS28, 28-joint Disease Activity Score; ESR, Erythrocyte sedimentation rate; GCs, glucocorticoids; HAQ-DI, Health Assessment Questionnaire Disability Index; IQR, interquartile range; MMP-3, Matrix metalloproteinase-3; MTX, methotrexate; RF, Rheumatoid factor; SDAI, Simplified Disease Activity Index


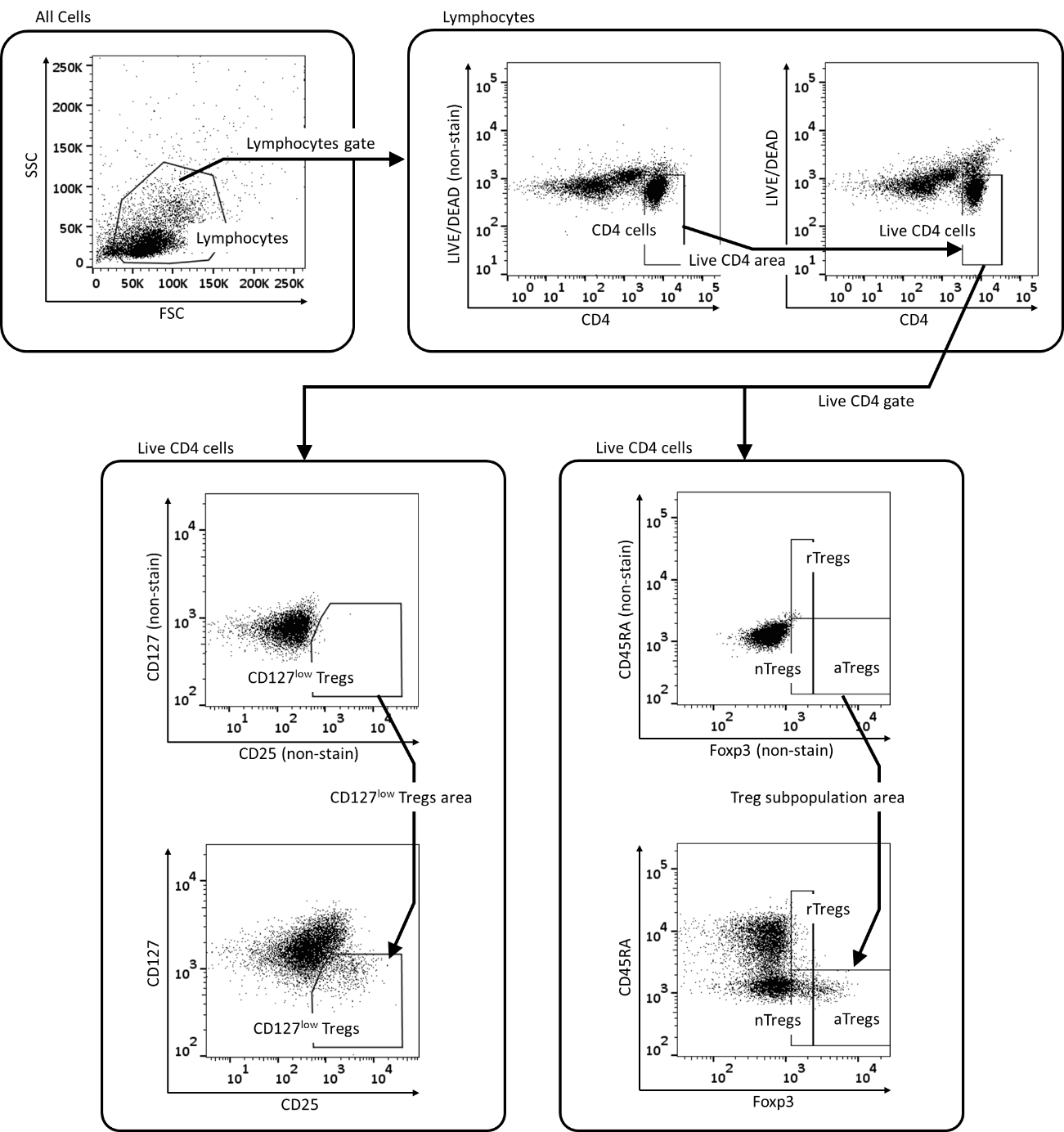


**Supplementary Figure S1. Gating strategy for flow cytometry analysis of Treg.**

PBMCs were stained with anti-CD4-APC/Cyanine7 (RPA-T4), anti-CD25-Brilliant Violet 421 (BC96), anti-CD45RA-Alexa Fluor 488 (HI100), and anti-CD127(IL-7Ra)-PerCP/Cyanine5.5 (A019D5), and anti-FOXP3-PE (259D) in the presence of LIVE/DEAD Fixable Aqua Stain. The gating strategy is shown by the arrows.


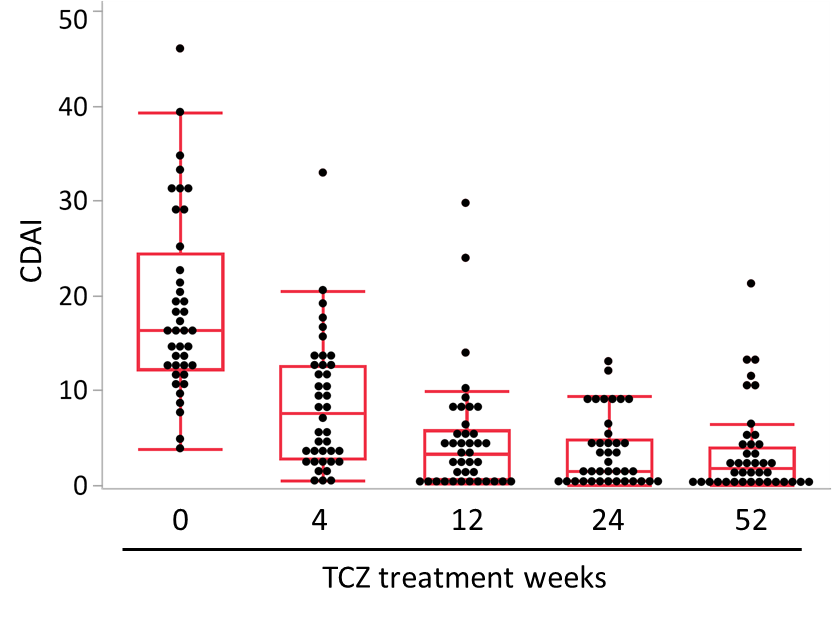


**Supplementary Figure S2. Change in disease activity score during TCZ treatment.**

Chronological change in CDAI from baseline by TCZ treatment is shown. The bottom, middle, and upper lines of the box plots correspond to the 25th, 50th, and 75th percentiles, respectively.


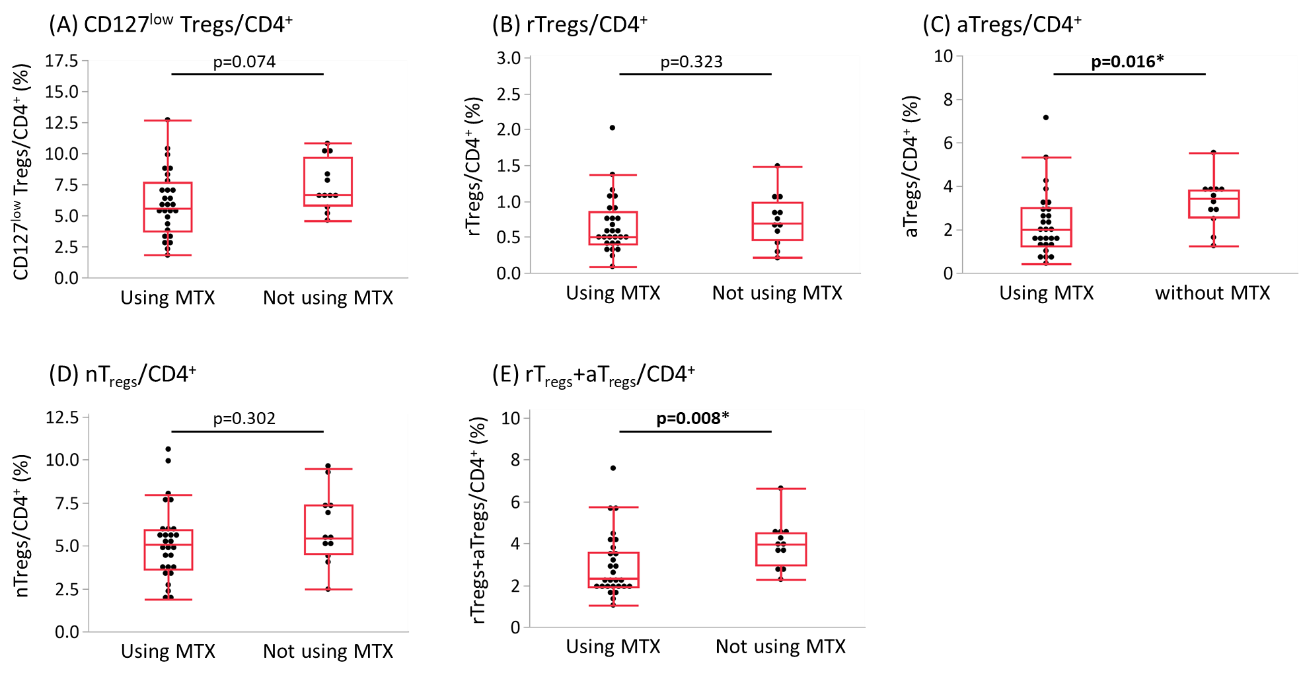


**Supplementary Figure S3. Comparison of the proportion of Treg subsets between RA patients using and not using MTX at baseline.**

Percentages of CD127^low^ Tregs (**A**), rTregs (**B**), aTregs (**C**), nTregs (**D**), and rTregs+aTregs (**E**) relative to CD4^+^ cells at Week 0 were compared between RA patients using MTX and RA patients not using MTX. The bottom, middle, and upper lines of the box plots correspond to the 25th, 50th, and 75th percentiles, respectively. Data were analyzed by Wilcoxon signed-rank test.

*, Statistically significant difference (*P* < 0.05).


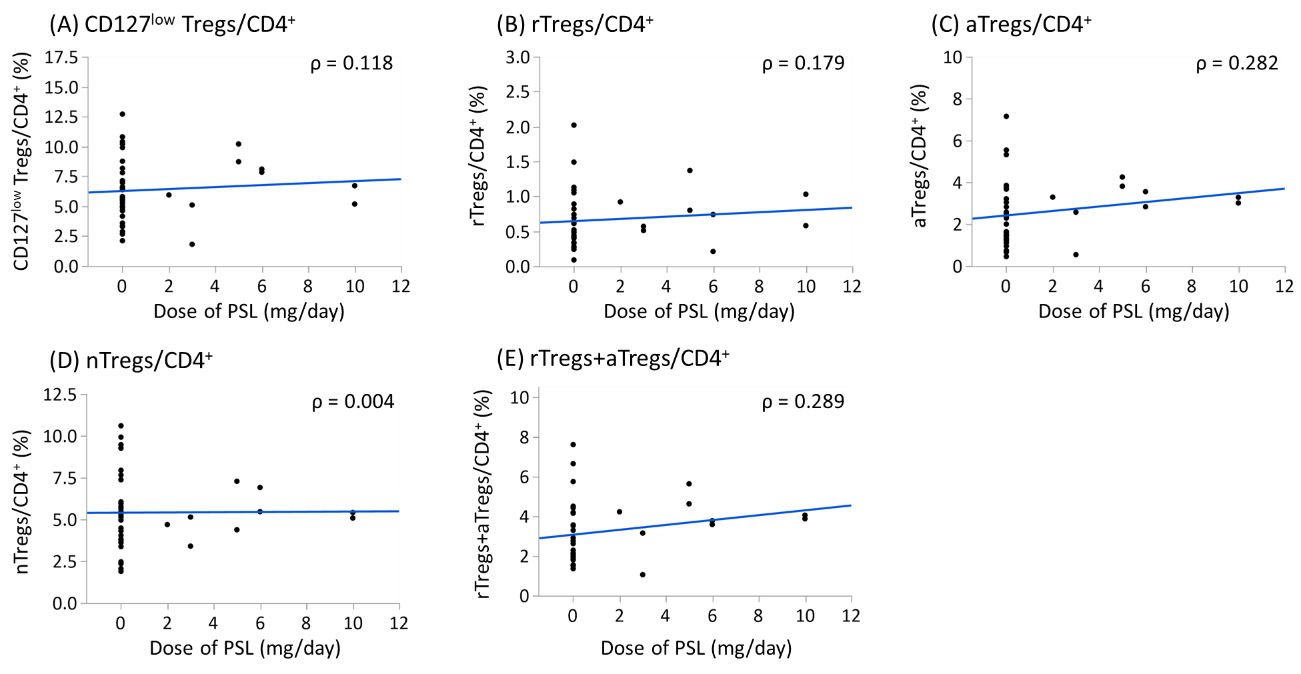


**Supplementary Figure S4. Correlations at baseline between the proportions of Treg subsets and the dose of PSL.**

The relationships at Week 0 between PSL dose and the proportions of CD127^low^ Tregs (**A**), rTregs (**B**), aTregs (**C**), nTregs (**D**), and rTregs+aTregs (**E**) relative to CD4^+^ cells are shown. Data were analyzed by Spearman rank correlation coefficient, and Spearman’s ρ values are provided. No significant correlation was found between PSL and any Treg subset at baseline.
